# Looking back to move forward: insights from retrospective cohort studies

**DOI:** 10.36416/1806-3756/e20250213

**Published:** 2025-07-22

**Authors:** Juliana Carvalho Ferreira, Cecilia M Patino

**Affiliations:** 1. Methods in Epidemiologic, Clinical, and Operations Research-MECOR-program, American Thoracic Society/Asociación Latinoamericana del Tórax, Montevideo, Uruguay.; 2. Divisão de Pneumologia, Instituto do Coração, Hospital das Clínicas Faculdade de Medicina, Universidade de São Paulo, São Paulo (SP) Brasil.; 3. Department of Preventive Medicine, Keck School of Medicine, University of Southern California, Los Angeles, CA, USA.

## PRACTICAL SCENARIO

Investigators were interested in estimating the incidence of noninvasive ventilation (NIV) failure in adult patients with acute respiratory failure and identifying the risk factors for failure. They performed a retrospective cohort study and examined the electronic medical records of 2,258 patients admitted to a 31-bed ICU in Brazil over one year and included 114 patients with acute respiratory failure who had received NIV support as the first line of treatment.^1^ They reported that the incidence of NIV failure was 41% and that predictors of NIV failure were male sex, infection as the primary cause of acute respiratory failure, and severity of illness at ICU admission. They also found that NIV failure was a risk factor for ICU mortality among those patients.

## WHAT ARE COHORT STUDIES?

Cohort studies are defined as observational studies in which participants at risk of developing one or more outcomes of interest are followed over time with the objective of describing the incidence of those outcomes and estimating their association with one or more exposures or predictors. At the beginning of follow-up, participants are free of the outcome(s) of interest. Exposures/predictors of interest are measured at baseline and periodically during follow-up, and patients are followed over time until the end of the study or in the occurrence of the outcome(s) or loss to follow-up.^2^

**Figure 1:**
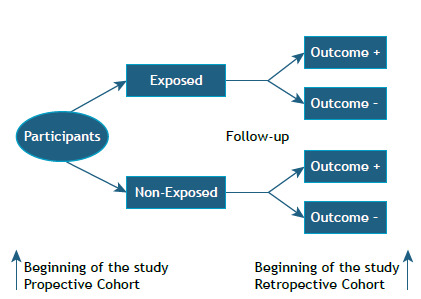
Structure of cohort studies.

Cohort studies are prospective when investigators plan the study and define the variables of interest before enrolling patients and follow them over time. Participants are assessed periodically for the occurrence of the outcome. The minimum duration of follow-up and periodicity of measurements depend on the type of outcome and on known or suspected latency between the exposure and the occurrence of the outcome. A cohort study is retrospective when follow-up of participants and outcomes have already occurred when the investigators start the study (Figure 1). Therefore, the study population has already been defined, measurements of the exposure and outcome have already been made by other professionals, and follow-up time has already taken place.

Prospective cohorts allow the investigator to measure variables of interest more accurately and completely, but they can be time consuming, expensive, and inappropriate when immediate results are warranted, given that investigators may need to follow patients over long periods of time. Retrospective studies take advantage of existing data, such as patient medical records, collected in the past for other purposes, thus shortening the duration of the study and decreasing measurement and personnel costs.

## ADVANTAGES AND DISADVANTAGES OF RETROSPECTIVE COHORTS

The main advantages of retrospective cohort studies are that they allow investigators to estimate the incidence rate of an outcome in an at-risk population and provide potential causes of outcomes of interest. Since the exposure is present before the outcome occurs, potential causation can be suspected, but not asserted, due to the risk that other unsuspected and/or unmeasured exposures may be the real risk factor for the occurrence of the outcome. 

Other advantages of retrospective cohorts include reduced cost and time when compared with prospective cohorts and clinical trials by providing a list of suspected risk factors for the outcomes of interest. In our practical scenario, the study was completed in two months, with minimal funding, whereas, if a prospective cohort had been chosen, the investigators would have needed to spend an entire year screening patients in the ICU daily to include the 114 patients and to be present in the ICU daily to collect demographic, ventilatory, and outcome data. 

The main disadvantages of retrospective cohort studies include the limited control that investigators have over the accuracy and completeness of the measurements, availability of important covariates that may also be associated with or predict the outcome, and biases, including selection bias, related to the population included in the study, and measurement bias, related to the fact that measurements were made before the investigators designed the study. For example, in our practical scenario, data related to the presence of relevant comorbidities such as COPD may have been missing or been inaccurate on the electronic medical records, which could have impacted the results. Thus, it is important to identify these issues when designing retrospective cohort studies. 

## KEY MESSAGES

1. Retrospective cohorts allow investigators to estimate the incidence of outcome(s) of interest in an at-risk population with reduced time and costs
2. Follow-up, measurement of the exposures, predictors, confounders, effect modifiers, and outcomes happened in the past, before the investigators have begun the study, and thus data is collected retrospectively
3. The main disadvantage of retrospective cohorts is the risk of inaccurate and incomplete data
